# Crystal structure of 7,8-benzocoumarin-4-acetic acid

**DOI:** 10.1107/S2056989015014103

**Published:** 2015-07-31

**Authors:** R. Ranga Swamy, Ramakrishna Gowda, K. V. Arjuna Gowda, Mahantesha Basanagouda

**Affiliations:** a Department of Physics, Sri Krishna Rajendra Silver Jubilee Institute of Technology, Bangalore 560 001, Karnataka, India; b Department of Physics, Govt. College for Women, Kolar 563 101, Karnataka, India; c Department of Physics, Govt. College for Women, Mandya 571 401, India; d Department of Chemistry, P.C. Jabin Science College, Hubli 580 031, Karnataka, India

**Keywords:** crystal structure, coumarin, acetic acid, hydrogen bonding

## Abstract

The fused-ring system in the title compound [systematic name: 2-(2-oxo-2*H*-benzo[*h*]chromen-4-yl)acetic acid], C_15_H_10_O_4_, is almost planar (r.m.s. deviation = 0.031 Å) and the C_ar_—C—C=O (ar = aromatic) torsion angle for the side chain is −134.4 (3)°. In the crystal, mol­ecules are linked by O—H⋯O hydrogen bonds, generating [100] *C*(8) chains, where the acceptor atom is the exocyclic O atom of the fused-ring system. The packing is consolidated by a very weak C—H⋯O hydrogen bond to the same acceptor atom. Together, these inter­actions lead to undulating (001) layers in the crystal.

## Related literature   

For a related structure and background to coumarins, see: Basanagouda *et al.* (2009 ▸). For the synthesis, see: Laskowski & Clinton (1950 ▸). 
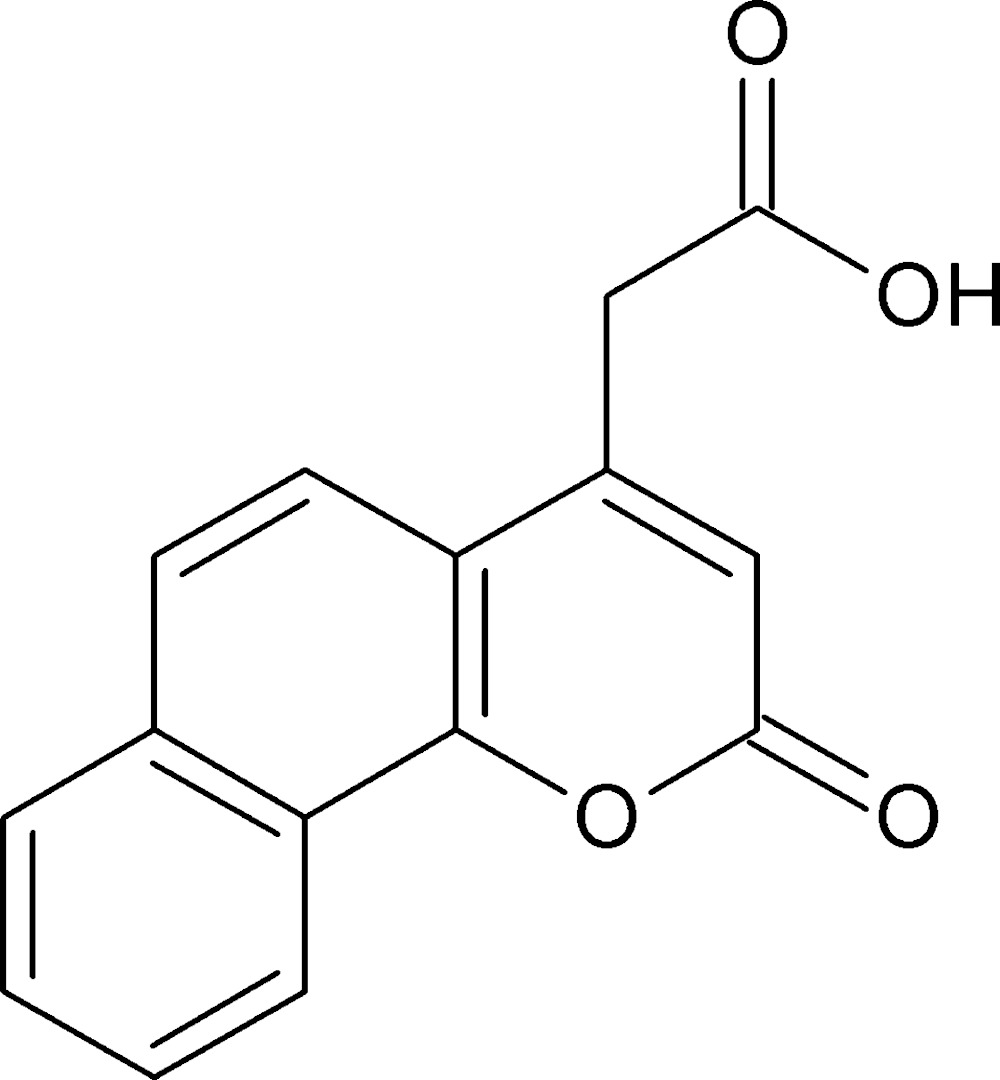


## Experimental   

### Crystal data  

C_15_H_10_O_4_
*M* *_r_* = 254.23
Orthorhombic, 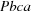

*a* = 13.4231 (4) Å
*b* = 8.9892 (3) Å
*c* = 18.8407 (6) Å
*V* = 2273.37 (12) Å^3^
*Z* = 8
Mo *K*α radiation
μ = 0.11 mm^−1^
*T* = 293 K
0.35 × 0.30 × 0.25 mm

### Data collection  

Bruker Kappa APEXII CCD diffractometer
Absorption correction: multi-scan (*SADABS*; Bruker, 2004 ▸) *T*_min_ = 0.961, *T*_max_ = 0.979
27493 measured reflections
2001 independent reflections
1356 reflections with *I* > 2σ(*I*)
*R*_int_ = 0.051

### Refinement  

*R*[*F* ^2^ > 2σ(*F* ^2^)] = 0.044
*wR*(*F* ^2^) = 0.138
*S* = 1.15
2001 reflections
173 parameters
H-atom parameters constrained
Δρ_max_ = 0.19 e Å^−3^
Δρ_min_ = −0.21 e Å^−3^

### 

Data collection: *APEX2* (Bruker, 2004 ▸); cell refinement: *SAINT* (Bruker, 2004 ▸); data reduction: *SAINT*; program(s) used to solve structure: *SIR92* (Altomare *et al.*, 1994 ▸); program(s) used to refine structure: *SHELXL2014* (Sheldrick, 2015 ▸); molecular graphics: *ORTEP-3 for Windows* (Farrugia, 2012 ▸); software used to prepare material for publication: *SHELXL2014*.

## Supplementary Material

Crystal structure: contains datablock(s) I, New_Global_Publ_Block. DOI: 10.1107/S2056989015014103/hb7471sup1.cif

Structure factors: contains datablock(s) I. DOI: 10.1107/S2056989015014103/hb7471Isup2.hkl

Supporting information file. DOI: 10.1107/S2056989015014103/hb7471Isup3.cml

**Fig. 1.**
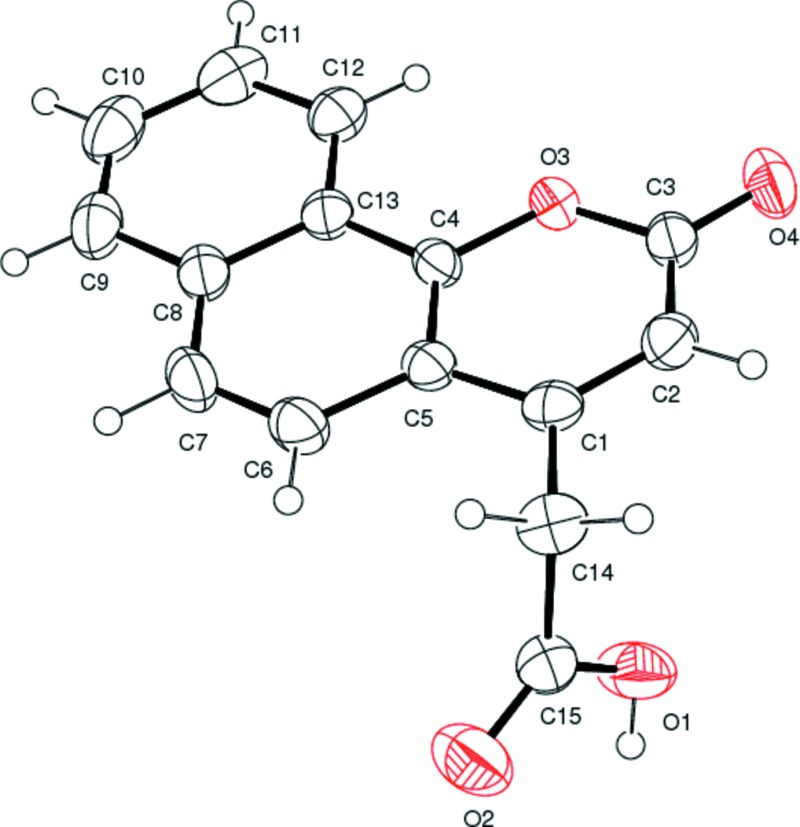
ORTEP diagram of molecule (I) with 40% probability displacement ellipsoids.

ORTEP . DOI: 10.1107/S2056989015014103/hb7471fig1.tif
*ORTEP* diagram of mol­ecule (I) with 40% probability displacement ellipsoids.

**Fig. 2.**
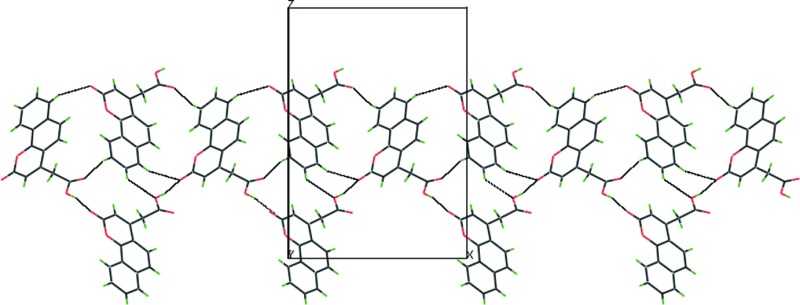
The packing diagram of (I)·The dotted lines indicate hydrogen bonds. All H atoms which are not in interactions have been omitted for clarity.

. DOI: 10.1107/S2056989015014103/hb7471fig2.tif
The packing diagram of (I)·The dotted lines indicate hydrogen bonds. All H atoms which are not in inter­actions have been omitted for clarity.

CCDC reference: 1415238

Additional supporting information:  crystallographic information; 3D view; checkCIF report

## Figures and Tables

**Table 1:** Hydrogen-bond geometry (, )

*D*H*A*	*D*H	H*A*	*D* *A*	*D*H*A*
O1H1O4^i^	0.82	1.84	2.626(3)	160
C14H14*A*O4^ii^	0.97	2.57	3.503(4)	161

Symmetry codes: (i) 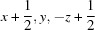; (ii) 
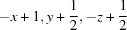
.

## supplementary crystallographic information

### S1. Experimental

A mixture of citric acid 2 (1 mol) and conc. sulfuric acid (32 ml) was stirred for half an hour, then the temperature was slowly raised during an interval of 10–15 min and as soon as the evolution of gas slackened, the flask was removed from the bath, allowed to stand for 15 min till the reaction mixture became clear and free from carbon monoxide bubbles; this was then cooled to 10°C. To this solution, 1-naphthol (1 mol) was added at 10°C, drop wise. After the addition, the reaction mixture was stirred at room temperature for 48 h. The reaction mixture was then poured onto crushed ice, the separated solid was filtered and dissolved in saturated sodium bicarbonate solution which on acidification gave the title compound (Laskowski *et al.* 1950). Colourless blocks were recrystallised from acetic acid solution at room temperature.

### S2. Refinement

All the H atoms in (I) were positioned geometrically and refined using a riding model with bond lengths 0.97 \%A (for methylene), 0.96 \%A (for methyl and 0.86 \%A (for amine). The *U*\ĩso\~(H) = 1.5*U*\~eq\~(C) for methyl and *U*\ĩso\~(H) = 1.2*U*\~eq\~(C) for all other carbon bound H atoms.

### Crystal data

**Table:**

C_15_H_10_O_4_	*D*_x_ = 1.486 Mg m^−^^3^
*M**_r_* = 254.23	Melting point: 463 K
Orthorhombic, *P**b**c**a*	Mo *K*α radiation, λ = 0.71073 Å
*a* = 13.4231 (4) Å	Cell parameters from 6795 reflections
*b* = 8.9892 (3) Å	θ = 2.6–27.0°
*c* = 18.8407 (6) Å	µ = 0.11 mm^−^^1^
*V* = 2273.37 (12) Å^3^	*T* = 293 K
*Z* = 8	Block, colourless
*F*(000) = 1056	0.35 × 0.30 × 0.25 mm

### Data collection

**Table:**

Bruker Kappa APEXII CCD diffractometer	2001 independent reflections
Radiation source: fine-focus sealed tube	1356 reflections with *I* > 2σ(*I*)
Graphite monochromator	*R*_int_ = 0.051
ω and φ scan	θ_max_ = 25.0°, θ_min_ = 2.2°
Absorption correction: multi-scan (*SADABS*; Bruker, 2004)	*h* = −15→14
*T*_min_ = 0.961, *T*_max_ = 0.979	*k* = −10→10
27493 measured reflections	*l* = −22→22

### Refinement

**Table:**

Refinement on *F*^2^	Hydrogen site location: inferred from neighbouring sites
Least-squares matrix: full	H-atom parameters constrained
*R*[*F*^2^ > 2σ(*F*^2^)] = 0.044	*w* = 1/[σ^2^(*F*_o_^2^) + (0.0513*P*)^2^ + 1.4242*P*] where *P* = (*F*_o_^2^ + 2*F*_c_^2^)/3
*wR*(*F*^2^) = 0.138	(Δ/σ)_max_ < 0.001
*S* = 1.15	Δρ_max_ = 0.19 e Å^−^^3^
2001 reflections	Δρ_min_ = −0.21 e Å^−^^3^
173 parameters	Extinction correction: *SHELXL2014* (Sheldrick, 2015), Fc^*^=kFc[1+0.001xFc^2^λ^3^/sin(2θ)]^-1/4^
0 restraints	Extinction coefficient: 0.0072 (15)

### Special details

**Table:**

Geometry. All e.s.d.'s (except the e.s.d. in the dihedral angle between two l.s. planes) are estimated using the full covariance matrix. The cell e.s.d.'s are taken into account individually in the estimation of e.s.d.'s in distances, angles and torsion angles; correlations between e.s.d.'s in cell parameters are only used when they are defined by crystal symmetry. An approximate (isotropic) treatment of cell e.s.d.'s is used for estimating e.s.d.'s involving l.s. planes.

### Fractional atomic coordinates and isotropic or equivalent isotropic displacement parameters (Å^2^)

**Table:**

	*x*	*y*	*z*	*U*_iso_*/*U*_eq_	
C1	0.61455 (18)	0.5555 (3)	0.36390 (13)	0.0399 (6)	
C2	0.53352 (18)	0.5222 (3)	0.32542 (13)	0.0437 (7)	
H2	0.5206	0.5762	0.2843	0.052*	
C3	0.46688 (18)	0.4072 (3)	0.34547 (14)	0.0425 (7)	
C4	0.56434 (16)	0.3698 (3)	0.44910 (12)	0.0352 (6)	
C5	0.63240 (16)	0.4753 (3)	0.42875 (13)	0.0373 (6)	
C6	0.71549 (19)	0.5000 (3)	0.47401 (15)	0.0491 (7)	
H6	0.7635	0.5697	0.4613	0.059*	
C7	0.7255 (2)	0.4236 (4)	0.53509 (15)	0.0538 (8)	
H7	0.7809	0.4412	0.5636	0.065*	
C8	0.65443 (18)	0.3177 (3)	0.55708 (13)	0.0434 (7)	
C9	0.6629 (2)	0.2399 (4)	0.62160 (15)	0.0567 (8)	
H9	0.7168	0.2582	0.6514	0.068*	
C10	0.5932 (3)	0.1385 (4)	0.64072 (16)	0.0649 (9)	
H10	0.5996	0.0882	0.6836	0.078*	
C11	0.5123 (2)	0.1087 (3)	0.59706 (16)	0.0579 (8)	
H11	0.4654	0.0381	0.6107	0.069*	
C12	0.5010 (2)	0.1822 (3)	0.53436 (14)	0.0466 (7)	
H12	0.4465	0.1618	0.5055	0.056*	
C13	0.57165 (17)	0.2887 (3)	0.51328 (12)	0.0377 (6)	
C14	0.6857 (2)	0.6737 (3)	0.33961 (16)	0.0508 (7)	
H14A	0.6521	0.7373	0.3056	0.061*	
H14B	0.7041	0.7347	0.3800	0.061*	
C15	0.7782 (2)	0.6133 (3)	0.30629 (14)	0.0461 (7)	
O1	0.75750 (14)	0.5102 (2)	0.25935 (11)	0.0661 (6)	
H1	0.8094	0.4790	0.2419	0.099*	
O2	0.86066 (15)	0.6547 (3)	0.31854 (12)	0.0770 (7)	
O3	0.48342 (11)	0.33699 (19)	0.40811 (9)	0.0407 (5)	
O4	0.39440 (14)	0.3648 (2)	0.31208 (10)	0.0597 (6)	

### Atomic displacement parameters (Å^2^)

**Table:**

	*U*^11^	*U*^22^	*U*^33^	*U*^12^	*U*^13^	*U*^23^
C1	0.0375 (13)	0.0406 (15)	0.0415 (15)	0.0035 (11)	0.0080 (11)	−0.0058 (12)
C2	0.0450 (14)	0.0481 (16)	0.0378 (14)	0.0073 (13)	0.0017 (12)	0.0011 (12)
C3	0.0388 (14)	0.0516 (16)	0.0373 (15)	0.0069 (13)	−0.0039 (12)	−0.0045 (13)
C4	0.0280 (12)	0.0415 (14)	0.0363 (14)	0.0034 (11)	−0.0024 (10)	−0.0093 (11)
C5	0.0331 (12)	0.0410 (14)	0.0378 (14)	0.0009 (11)	0.0020 (10)	−0.0089 (12)
C6	0.0383 (14)	0.0575 (18)	0.0514 (17)	−0.0072 (13)	−0.0008 (12)	−0.0126 (15)
C7	0.0418 (16)	0.070 (2)	0.0491 (17)	0.0035 (14)	−0.0135 (12)	−0.0190 (16)
C8	0.0407 (14)	0.0504 (16)	0.0392 (15)	0.0126 (13)	−0.0036 (12)	−0.0106 (13)
C9	0.0597 (18)	0.070 (2)	0.0404 (17)	0.0230 (17)	−0.0100 (13)	−0.0061 (15)
C10	0.081 (2)	0.070 (2)	0.0435 (17)	0.0267 (19)	0.0038 (17)	0.0085 (16)
C11	0.068 (2)	0.0504 (18)	0.0547 (19)	0.0103 (15)	0.0165 (16)	0.0071 (15)
C12	0.0495 (15)	0.0457 (16)	0.0445 (16)	0.0037 (13)	0.0046 (13)	−0.0031 (14)
C13	0.0370 (13)	0.0408 (14)	0.0353 (14)	0.0081 (11)	0.0010 (11)	−0.0061 (12)
C14	0.0524 (16)	0.0466 (16)	0.0535 (17)	−0.0014 (13)	0.0087 (13)	−0.0011 (14)
C15	0.0453 (16)	0.0492 (17)	0.0438 (16)	−0.0035 (14)	0.0011 (12)	0.0035 (14)
O1	0.0475 (11)	0.0796 (15)	0.0714 (14)	−0.0079 (10)	0.0161 (10)	−0.0237 (13)
O2	0.0480 (12)	0.0937 (18)	0.0894 (17)	−0.0085 (12)	−0.0036 (11)	−0.0217 (14)
O3	0.0328 (9)	0.0511 (11)	0.0381 (10)	−0.0020 (8)	−0.0040 (7)	−0.0016 (8)
O4	0.0484 (11)	0.0761 (15)	0.0545 (12)	−0.0047 (10)	−0.0200 (9)	0.0020 (11)

### Geometric parameters (Å, º)

**Table:**

C1—C2	1.341 (3)	C8—C13	1.408 (3)
C1—C5	1.439 (3)	C9—C10	1.355 (4)
C1—C14	1.500 (4)	C9—H9	0.9300
C2—C3	1.419 (4)	C10—C11	1.389 (4)
C2—H2	0.9300	C10—H10	0.9300
C3—O4	1.220 (3)	C11—C12	1.362 (4)
C3—O3	1.357 (3)	C11—H11	0.9300
C4—O3	1.365 (3)	C12—C13	1.404 (4)
C4—C5	1.372 (3)	C12—H12	0.9300
C4—C13	1.416 (3)	C14—C15	1.494 (4)
C5—C6	1.421 (3)	C14—H14A	0.9700
C6—C7	1.347 (4)	C14—H14B	0.9700
C6—H6	0.9300	C15—O2	1.190 (3)
C7—C8	1.410 (4)	C15—O1	1.311 (3)
C7—H7	0.9300	O1—H1	0.8200
C8—C9	1.407 (4)		
			
C2—C1—C5	118.8 (2)	C10—C9—H9	119.7
C2—C1—C14	120.6 (2)	C8—C9—H9	119.7
C5—C1—C14	120.5 (2)	C9—C10—C11	120.8 (3)
C1—C2—C3	122.0 (2)	C9—C10—H10	119.6
C1—C2—H2	119.0	C11—C10—H10	119.6
C3—C2—H2	119.0	C12—C11—C10	120.5 (3)
O4—C3—O3	115.7 (2)	C12—C11—H11	119.8
O4—C3—C2	126.4 (3)	C10—C11—H11	119.8
O3—C3—C2	117.9 (2)	C11—C12—C13	120.1 (3)
O3—C4—C5	121.4 (2)	C11—C12—H12	120.0
O3—C4—C13	115.3 (2)	C13—C12—H12	120.0
C5—C4—C13	123.3 (2)	C12—C13—C8	119.6 (2)
C4—C5—C6	117.6 (2)	C12—C13—C4	123.1 (2)
C4—C5—C1	118.2 (2)	C8—C13—C4	117.4 (2)
C6—C5—C1	124.2 (2)	C15—C14—C1	113.6 (2)
C7—C6—C5	120.7 (3)	C15—C14—H14A	108.8
C7—C6—H6	119.6	C1—C14—H14A	108.8
C5—C6—H6	119.6	C15—C14—H14B	108.8
C6—C7—C8	121.9 (2)	C1—C14—H14B	108.8
C6—C7—H7	119.1	H14A—C14—H14B	107.7
C8—C7—H7	119.1	O2—C15—O1	123.3 (3)
C9—C8—C13	118.6 (3)	O2—C15—C14	125.3 (3)
C9—C8—C7	122.3 (3)	O1—C15—C14	111.3 (2)
C13—C8—C7	119.1 (2)	C15—O1—H1	109.5
C10—C9—C8	120.5 (3)	C3—O3—C4	121.5 (2)
			
C5—C1—C2—C3	−2.2 (4)	C9—C10—C11—C12	−0.6 (4)
C14—C1—C2—C3	177.5 (2)	C10—C11—C12—C13	0.2 (4)
C1—C2—C3—O4	−176.3 (3)	C11—C12—C13—C8	0.6 (4)
C1—C2—C3—O3	4.5 (4)	C11—C12—C13—C4	−179.6 (2)
O3—C4—C5—C6	−178.7 (2)	C9—C8—C13—C12	−0.9 (4)
C13—C4—C5—C6	1.8 (3)	C7—C8—C13—C12	179.3 (2)
O3—C4—C5—C1	2.3 (3)	C9—C8—C13—C4	179.2 (2)
C13—C4—C5—C1	−177.1 (2)	C7—C8—C13—C4	−0.5 (3)
C2—C1—C5—C4	−1.3 (3)	O3—C4—C13—C12	−0.4 (3)
C14—C1—C5—C4	179.1 (2)	C5—C4—C13—C12	179.1 (2)
C2—C1—C5—C6	179.9 (2)	O3—C4—C13—C8	179.4 (2)
C14—C1—C5—C6	0.2 (4)	C5—C4—C13—C8	−1.1 (3)
C4—C5—C6—C7	−1.0 (4)	C2—C1—C14—C15	−101.9 (3)
C1—C5—C6—C7	177.9 (2)	C5—C1—C14—C15	77.8 (3)
C5—C6—C7—C8	−0.6 (4)	C1—C14—C15—O2	−134.4 (3)
C6—C7—C8—C9	−178.4 (3)	C1—C14—C15—O1	47.6 (3)
C6—C7—C8—C13	1.3 (4)	O4—C3—O3—C4	177.3 (2)
C13—C8—C9—C10	0.5 (4)	C2—C3—O3—C4	−3.4 (3)
C7—C8—C9—C10	−179.7 (3)	C5—C4—O3—C3	0.1 (3)
C8—C9—C10—C11	0.3 (4)	C13—C4—O3—C3	179.6 (2)

### Hydrogen-bond geometry (Å, º)

**Table:**

*D*—H···*A*	*D*—H	H···*A*	*D*···*A*	*D*—H···*A*
O1—H1···O4^i^	0.82	1.84	2.626 (3)	160
C14—H14*A*···O4^ii^	0.97	2.57	3.503 (4)	161

Symmetry codes: (i) *x*+1/2, *y*, −*z*+1/2; (ii) −*x*+1, *y*+1/2, −*z*+1/2.
