# Supplementary figures and images for: Crystal structure of 7,8-benzocoumarin-4-acetic acid
Source: Acta Crystallogr E Crystallogr Commun. 2015 Jul 31;71(Pt 8):o617–8. doi: 10.1107/S2056989015014103 (PMC4571427; doi:10.1107/S2056989015014103)

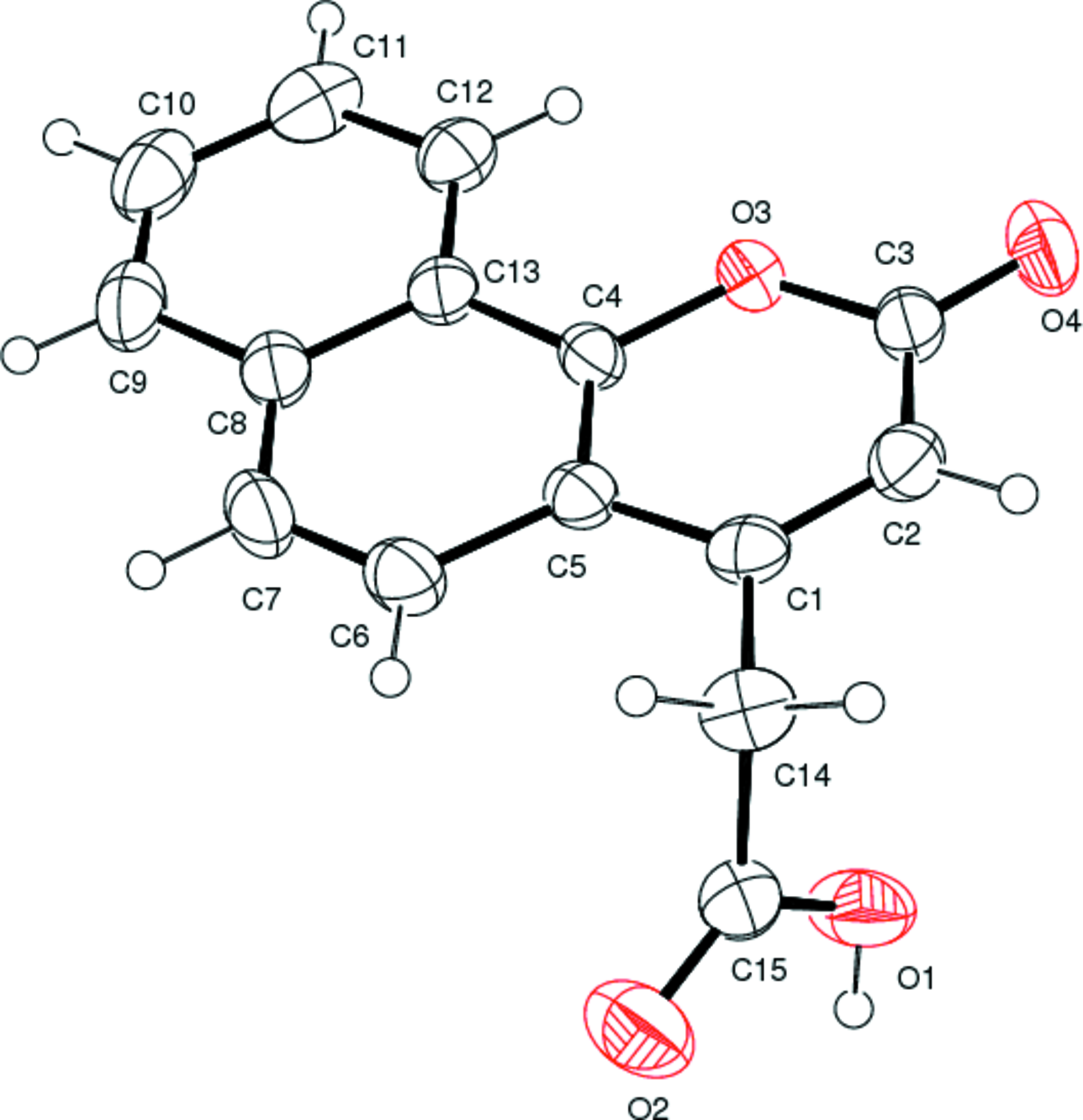

Supplement: Supplementary file 4 [file e-71-0o617-fig1.tif]

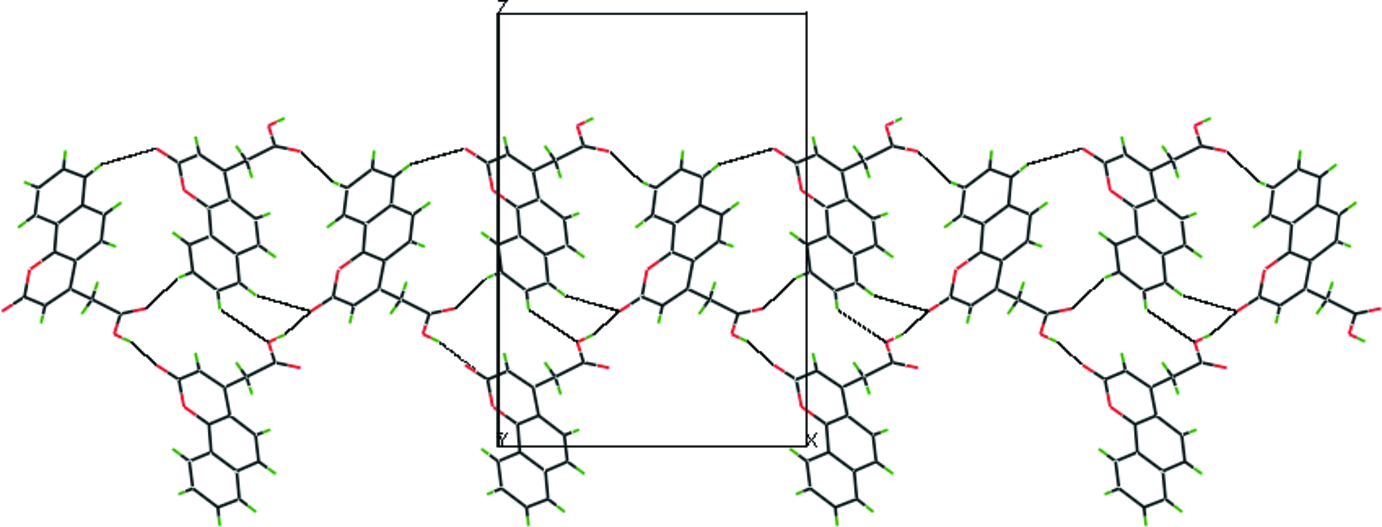

Supplement: Supplementary file 5 [file e-71-0o617-fig2.tif]
